# Helicopter Turboshaft Engines’ Neural Network System for Monitoring Sensor Failures

**DOI:** 10.3390/s25040990

**Published:** 2025-02-07

**Authors:** Serhii Vladov, Łukasz Ścisło, Nina Szczepanik-Ścisło, Anatoliy Sachenko, Tomasz Perzyński, Viktor Vasylenko, Victoria Vysotska

**Affiliations:** 1 Kharkiv National University of Internal Affairs, 27, L. Landau Avenue, 61080 Kharkiv, Ukraine; serhii.vladov@univd.edu.ua (S.V.); vasylenko_viktor@ukr.net (V.V.); 2 Faculty of Electrical and Computer Engineering, Cracow University of Technology, 24, Warszawska, 31-155 Cracow, Poland; 3 Faculty of Environmental Engineering and Energy, Cracow University of Technology, 24, Warszawska, 31-155 Cracow, Poland; nina.szczepanik@pk.edu.pl; 4 CERN, European Organization for Nuclear Research, 1, Esplanade des Particules, 1211 Geneva 23, Switzerland; 5 Research Institute for Intelligent Computer Systems, West Ukrainian National University, 11, Lvivska Street, 46009 Ternopil, Ukraine; as@wunu.edu.ua; 6 Faculty of Transport, Electrical Engineering and Computer Science, Casimir Pulaski Radom University, 26-600 Radom, Poland; 7 Information Systems and Networks Department, Lviv Polytechnic National University, 12, Bandera Street, 79013 Lviv, Ukraine; victoria.a.vysotska@lpnu.ua; 8 Institute of Computer Science, Osnabrück University, 1, Friedrich-Janssen-Street, 49076 Osnabrück, Germany

**Keywords:** sensor failures, neural network system, helicopter turboshaft engines, sensors, recurrent layers, approximation, anomaly detection

## Abstract

An effective neural network system for monitoring sensors in helicopter turboshaft engines has been developed based on a hybrid architecture combining LSTM and GRU. This system enables sequential data processing while ensuring high accuracy in anomaly detection. Using recurrent layers (LSTM/GRU) is critical for dependencies among data time series analysis and identification, facilitating key information retention from previous states. Modules such as SensorFailClean and SensorFailNorm implement adaptive discretization and quantisation techniques, enhancing the data input quality and contributing to more accurate predictions. The developed system demonstrated anomaly detection accuracy at 99.327% after 200 training epochs, with a reduction in loss from 2.5 to 0.5%, indicating stability in anomaly processing. A training algorithm incorporating temporal regularization and a combined optimization method (SGD with RMSProp) accelerated neural network convergence, reducing the training time to 4 min and 13 s while achieving an accuracy of 0.993. Comparisons with alternative methods indicate superior performance for the proposed approach across key metrics, including accuracy at 0.993 compared to 0.981 and 0.982. Computational experiments confirmed the presence of the highly correlated sensor and demonstrated the method’s effectiveness in fault detection, highlighting the system’s capability to minimize omissions.

## 1. Introduction

In modern aviation engineering, reliability and operational safety in helicopter turboshaft engines (TEs) require continuous monitoring and improvement. Sensors installed in helicopter TE systems play a vital role in monitoring their technical condition, enabling the timely detection of anomalies and malfunctions [1,2]. However, sensor data (telemetry) are subject to various external and internal influences [3], potentially leading to measurement errors or data loss. In this context, the development of intelligent control and diagnostic systems capable of identifying sensor anomalies and restoring lost data has become increasingly relevant. One promising solution involves implementing neural network control systems to analyze incoming sensor signals, detect deviations from normal conditions, and predict potential failures in helicopter TEs [4,5,6]. Based on deep learning methods, such systems can adapt to changing engine operating conditions and learn from historical data.

The development of helicopter TE sensor failure neural network control systems is driven by the need to enhance automation in diagnostics, improve the aviation systems reliability, and minimize the risks associated with engine failures. Using modern machine learning technologies to analyze sensor data opens new possibilities for creating more resilient and efficient control systems, significantly enhancing the safety and operational effectiveness of helicopters.

Numerous recent studies have focused on developing intelligent systems for monitoring and diagnosing helicopter TE sensors. Many of these systems are based on neural network approaches, which have demonstrated high efficiency in detecting faults and the capability of systems to predict technical conditions [7,8]. One primary research direction is the use of recurrent neural networks, particularly Long Short-Term Memory (LSTM) networks, for analyzing sensor time series data [9,10,11]. These models can identify hidden patterns in data and accurately predict anomalous events.

Hybrid models, combining neural networks with fuzzy logic methods and decision support systems, are also actively researched [12,13,14]. Such approaches account for uncertainties and noise in the data, which is crucial for aviation systems, where measurement accuracy can be affected by external factors like vibrations or changing operating conditions. Research [15,16] indicates that hybrid neural network systems significantly improve diagnostic reliability and accuracy compared to traditional methods, such as statistical models and simple threshold-based anomaly detection techniques.

Innovative frameworks such as optimized extreme learning machines (ELMs) [17] demonstrate high speed and reliability in structural damage identification. Their application in helicopter TE diagnostics enables accurate damage detection while minimizing computational costs and data processing time [18]. In the equipment condition prediction field, the integration of convolutional neural networks (CNNs) with bidirectional LSTM models (DBLSTMs) is promising [19,20]. Such approaches are used, for example, for the long-term prediction of a component’s remaining service life [21], including batteries [22], indicating their potential in complex systems in aviation technical conditions for predicting and diagnosing tasks. These achievements provide the basis for the development of adaptive training algorithms capable of taking into account changing operating conditions and data uncertainties, which are especially important for complex aviation systems.

Additionally, adaptive training algorithms contribute development significantly to sensor monitoring systems advancements [23,24]. These algorithms can update parameters in real time, allowing for self-learning and adaptation to changes in engine performance or component behaviour [25,26,27], making them more effective in dynamic operating environments. Comparative studies show that helicopter TE adaptive neural network sensor monitoring systems deliver faster and more accurate results, which are crucial for preventing critical failures and enhancing flight safety.

Despite the progress in helicopter TE development for neural network-based sensor monitoring systems [28,29], essential challenges that limit their application remain. The primary challenge is the model’s insufficient robustness to noise and interference caused by vibrations, temperature changes, and pressure fluctuations. It can lead to false alarms or missed faults in real-world conditions. Additionally, many current systems overlook helicopter TE physical models, relying solely on sensor data, which reduces diagnostic accuracy, especially in non-standard operating modes. Integrating physical models could significantly enhance prediction accuracy.

Thus, new approaches that combine neural network technologies with hypothesis generation and validation methods for sensor data correlation and plausibility are needed based on helicopter TE physical models and design features. This would improve diagnostic accuracy and reliability, especially in noisy environments, and provide a deeper understanding of engine processes, critical for enhancing helicopter operations safety and efficiency.

**The research aim** is to develop a method for detecting helicopter TE sensor faults and identifying telemetry segments with errors using neural network technologies, which can efficiently recognize and classify situations even with incomplete or ambiguous data. Computation speed is increased through the use of modern cluster setups. The **research object** is helicopter TE sensor fault detection systems, their processes, and their characteristics. The **research subject** involves methods and tools for detecting helicopter TE sensor faults during flight operations. The proposed monitoring system operates as follows:

- All sensor readings are recorded in a database;
- A second assessment is conducted;
- A model of the current state of the monitored equipment is built;
- Simulated values are stored in the database;
- Information regarding detected faults is relayed to the human operator.

## 2. Materials and Methods

### 2.1. Statement of the Problem

This article addresses the sensor system monitoring challenge under interference by proposing and testing hypotheses on the correlation and plausibility of the readings based on helicopter TE physical models and design features. The data for the research, as well as the necessary equipment (computer equipment, helicopter TE) were obtained from the Ministry of Internal Affairs of Ukraine (Kyiv, Ukraine). The complexity of the helicopter TE detecting sensor failures and malfunctions arises from the lack of telemetry data containing examples of various faults and the inability to conduct large-scale experiments. The only viable approach is formulating and testing hypotheses using heuristic models considering helicopter TE design characteristics and the positioning of control elements. An engine transition to operational modes (nominal, cruise I, cruise II, and emergency) typically changes other sensor readings. Assuming that functional sensors exhibit stable correlation in their readings, a sharp decrease in such correlation during engine operation may signal a potential fault. The monitoring is based on the following hypotheses:

**Hypothesis** **1.** *Due to the helicopter TE design features, functional sensors exhibit stable correlations with each other.*

**Hypothesis** **2.** *Anomalies contradicting the helicopter TE design features and models are categorized as faults or interferences.*

Here, *X_i_*(*t*) represents the *i*-th functional sensor readings at time *t*, and *X_j_*(*t*) represents the readings of another functional *j*-th sensor. Hypothesis 1 states that functional sensors maintain a stable correlation between their readings.

Corr(*X_i_*(*t*), *X_j_*(*t*)) ≈ *ρ_ij_*, ∀*i*, *j* ∈ {1, 2,…, *N*}, *t* ∈ *T*, (1)

where *ρ_ij_* represents the correlation coefficient between the sensor *i*-th and sensor *j*-th readings, *N* is the sensor’s total number, and *T* is the time interval.

Fault monitoring can be performed based on the sharp change condition in the correlation coefficient between sensors. If a sudden change in correlation is observed between the sensor pair *i* and *j*, the following is obtained:

ΔCorr(*X_i_*(*t*), *X_j_*(*t*)) ∣Corr(*X_i_*(*t*), *X_j_*(*t*)) − *ρ_ij_*∣ > *ϵ*, (2)

where *ϵ* is the threshold value at which a malfunction or failure is assumed.

Suppose the engine transitions from one operational mode to another (nominal to emergency mode). In that case, the sensor reading’s dependency on the operational mode *R_k_* can be assumed (where *k* represents the mode number):

*X_i_*(*t*) = *f_i_*(*R_k_*(*t*)) + *ε_i_*(*t*), (3)

where *f_i_*(*R_k_*(*t*)) represents a function that describes the sensor reading’s dependency on the operational mode, and *ε_i_*(*t*) represents noise or measurement error.

Hypothesis 2 asserts that anomalies not conforming to the helicopter TE model or design features are considered faults or interferences. To address this, an error function *δ_i_*(*t*) can be introduced for the *i*-th sensor, indicating the actual data’s deviation from expected values:

*δ_i_*(*t*) = |*X_i_*(*t*) − *f_i_*(*R_k_*(*t*))| > *α*, (4)

where *α* is the acceptable error; if the error *δ_i_*(*t*) exceeds the threshold *α*, a failure is recorded.

The total error use is proposed to evaluate the overall condition across a sensor group:

(5) $$∆\left(t\right)=\sum_{i=1}^{N}\delta_{i}\left(t\right).$$

If Δ(*t*) > *β*, where *β* is the threshold value, it is considered that a malfunction has occurred in the sensor system.

### 2.2. Proposed Method

#### 2.2.1. Development of the First Hypothesis Testing Method

To test the first hypothesis, a programme was developed that preprocessed the readings from helicopter TE sensors to check for a pairwise presence and triple correlations between the sensor readings. Data from six sensors measuring the following parameters were used: the gas-generator rotor r.p.m. (*n_TC_*), free turbine rotor speed (*n_FT_*), gas temperature before the compressor turbine ($T_{G}^{*}$), oil temperature at the engine inlet (*T_oil_*), oil pressure at the engine outlet (*P_oil_*), and main rotor speed (*n_rs_*). Based on the recorded sensor readings, all possible pair (*a*, *b*) and triple (*a*, *b*, *c*) combinations of *k* sensors were generated. The pairwise number and triple combinations are calculated as follows:

(6) $$C_{k}^{2}=\frac{k!}{2\cdot \left(k-2\right)},\;C_{k}^{3}=\frac{k!}{3\cdot \left(k-3\right)}.$$

To enhance signal quality, an adaptive discretization method is applied as follows:

(7) $$D\left(t_{i}\right)=\frac{1}{1+\gamma \cdot Var\left(x_{i}\right)},$$

where Var(*x_i_*) is the variance in the *t_i_* vicinity, and *γ* is the adaptive discretization coefficient.

The *x_i_* values quantization is performed to reduce noise and improve measurement accuracy:

(8) $$Q\left(x_{i}\right)=round\left(\frac{x_{i}-\min X}{∆}\right)\cdot ∆+\min X,$$

where round (•) is the rounding function, and Δ is the quantization step chosen based on signal statistics.

Wavelet transformation is applied to detect hidden patterns in the data. Appropriate basis wavelets *ψ*(*t*) are selected for signal decomposition. Discrete wavelet transformation (DWT) is applied to the signals to obtain their time–frequency representation:

(9) $$W\left(t,\tau \right)=\int_{-\infty }^{\infty }x\left(u\right)\cdot \psi \left(\frac{u-t}{\tau }\right)du.$$

where *x*(*u*) is the original signal and $\psi \left(\frac{u-t}{\tau }\right)$ is the basis of the wavelet with scale *τ* and shift *t*.

The obtained wavelet coefficients detect hidden patterns and anomalies in the data. Specifically, abnormal regions are identified when the wavelet coefficient values exceed a certain threshold. Correlation coefficients are computed for each sensor combination using both traditional methods and methods based on the enhanced data:

1. The rank difference di is determined for the comparable values of each pair.
2. The rank correlation coefficients are calculated using the following expression:
  (10) $$r_{j}=1-\frac{6\cdot \sum_{i=1}^{p}d_{i}^{2}}{p\cdot \left(p^{2}-1\right)},$$
  where $\sum_{i=1}^{p}d_{i}^{2}$ represents the squared rank difference sum *s*, and *p* is the paired observations number.
3. A sliding window is applied to analyze the data time series and calculate correlation coefficients at each window position. The window size is varied, and optimization methods are employed to determine the optimal window size that maximizes the average correlation coefficient for the combination. The arithmetic mean correlation is calculated as follows:
  (11) $$\bar{r}=\frac{1}{S}\cdot \sum_{j=1}^{S}r_{j},$$
  where *S* represents the number of window positions, and *r_j_* denotes the correlation coefficient for the *j*-th position.

The adaptive discretization and quantization implementation, combined with wavelet transformation, significantly enhances signal quality and reveals hidden patterns. The results of Hypothesis 1 with experimental verification are presented in Section 3.2.

#### 2.2.2. Development of the Second Hypothesis Testing Method

When addressing the task of fault detection using the parameter of the gas-generator rotor r.p.m. *n_TC_*, it is assumed that a measurement is recorded for the *n_TC_* parameter *x_i_* at time *t_i_*, creating a data array **X** = {*x*_1_, *x*_2_, …, *x_n_*}, where *n* represents the total number of the readings, which may include false changes in range due to noise.

Initially, adaptive discretization is applied to enhance signal quality (7), followed by the quantization of values *x_i_* to reduce noise and improve measurement accuracy (8). Subsequently, the actual value is calculated using a degree *d* of the polynomial that approximates the helicopter TE’s true characteristics while ignoring sensor faults:

*P*(*t*) = *a*_0_ + *a*_1_·*t* + *a*_2_·*t*^2^ + ⋯ + *a_d_*·*t^d^*, (12)

where *a*_0_, *a*_1_, …, *a_d_* represent the polynomial coefficients.

The coefficients *a_j_* are determined using the least squares method, expressed as follows:

(13) $$\underset{a_{0},a_{1},\ldots ,a_{d}}{\min}\sum_{i=1}^{n}{\left(x_{i}-P\left(t_{i}\right)\right)}^{2}.$$

The assumption regarding a fault is based on a significant deviation between measured data *x_i_* and approximated values *P*(*t_i_*). For instance, a sharp change in readings followed by a return to the original value may indicate interference or a fault, as this does not align with the helicopter TE operational dynamics and the helicopter flight. Such anomalous data must be identified and corrected. The following expression can be used to assess deviations:

(14) $${Dev}_{i}=\left|x_{i}-P\left(t_{i}\right)\right|.$$

The use of the polynomial for approximating readings enables the identification and exclusion of faults caused by interference. The challenge in data processing and polynomial selection lies in the outlier’s presence, which must be accounted for in the polynomial coefficients. The polynomial degree is determined through a measured and reconstructed data stepwise approximation.

The algorithm’s first step assigns a specific weight to all readings. This step identifies moments in time (readings) where actual data significantly deviate from the reconstructed polynomial. Readings with sharp changes are assigned a lower weight to minimize their impact on the polynomial:

(15) $$w_{i}=\frac{1}{1+k\cdot {Dev}_{i}},$$

where *k* is the sensitivity coefficient. Based on the new weights, the polynomial is re-selected as follows:

(16) $$\underset{a_{0},a_{1},\ldots ,a_{d}}{\min}\sum_{i=1}^{n}w_{i}\cdot {\left(x_{i}-P\left(t_{i}\right)\right)}^{2}.$$

In the second step, deviation analysis is conducted. A threshold value for deviation Δ is introduced, determined by the helicopter TE design features. The data are considered anomalous if the deviation exceeds the threshold value Δ (*Dev_i_* > Δ).

In the third step, the points have redistributed weights, and the polynomial is refitted. This process enhances the accuracy of sensor reading interpolation.

The results of Hypothesis 2 with experimental verification are presented in Section 3.2.

### 2.3. Development of a Neural Network System

**Figure 1:**
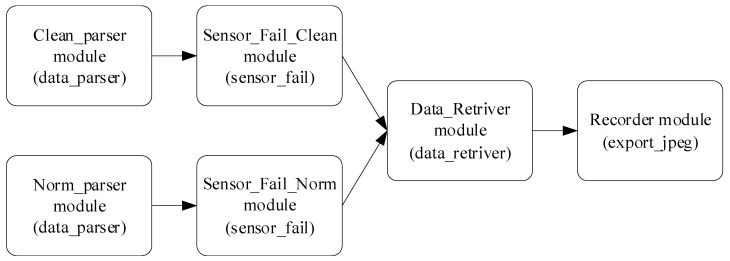
The proposed algorithm block diagram.

The research presents an algorithm for processing input data (see Figure 1), which enables sensor readings and correlations to be predicted based on known data [30,31]. Fault locations are identified by comparing actual and predicted correlation values. If the prediction error exceeds a specified threshold, a fault is recorded. The proposed input data processing algorithm consists of the following:

1. The clean_parser module (data_parser) sends telemetry data intended for smoothing to the Sensor_Fail_Clean module (sensor_fail) in the Input channel.
2. The norm_parser module (data_parser) sends telemetry data designated for normalization to the SensorFailNorm module (sensor_fail) in the Input channel.
3. The Sensor_Fail_Clean module (sensor_fail) smooths data, performs adaptive discretization and quantization and sends the results to the Data_Retriever module (data_retriver) in the Input_Train channel.
4. The Sensor_Fail_Norm module (sensor_fail) normalizes telemetry data and sends them to the Data_Retriever module (data_retriver) in the Input_Detect channel.
5. The Data_Retriever module (data_retriver) performs the following:
  - Determines sensor groups with maximum correlation based on data from the Input_Train channel;
  - Computes correlation values from the Input_Detect channel;
  - Identifies prediction errors in correlations.
6. The Recorder module (export_jpeg) receives graphical results from the Input channel and saves them.

**Figure 2:**
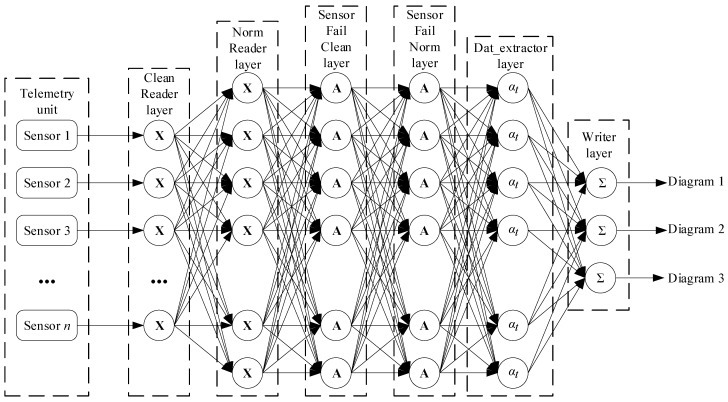
The proposed neural network structural diagram.

To implement this algorithm, the research proposes a recurrent neural network (RNN) utilizing LSTM (Long Short-Term Memory) [32] and GRU (Gated Recurrent Unit) [33] mechanisms, which represent enhanced versions of standard RNNs (Figure 2). The main idea behind this architecture is its ability to efficiently process temporal data sequences, making it ideally suited for analyzing telemetry data and forecasting correlations.

In the proposed neural network, input layers receive data (Clean Parser and Norm Parser), where recurrent layers process the data by performing smoothing, normalization, and correlation analysis (Sensor_Fail_Clean and Sensor_Fail_Norm); extraction layers identify correlations and errors (Data_Retriver); and the output layer stores the resulting diagrams (Recorder).

The Clean Parser layer (data_parser) is the input layer responsible for receiving telemetry data that require smoothing. The data are transmitted to the Sensor_Fail_Clean module through the Input channel. The network’s input layer accepts data for smoothing and forwards them for adaptive processing.

The Norm Parser layer (data_parser) is the input layer, which operates similarly, but the data are directed to normalization in the Sensor_Fail_Norm module through the Input channel. Here, the input layer receives data for normalization and forwards them for further processing.

The Sensor_Fail_Clean layer (sensor_fail) is the recurrent layer + adaptive processing, which processes telemetry data for smoothing using adaptive discretization and quantization, then transmits the results to the next module via the Input_Train channel.

The Sensor_Fail_Norm layer (sensor_fail) is the recurrent layer + normalization, which normalizes telemetry data. Like the previous module, it is a recurrent layer with added data normalization functionality, passing the data to the next module through the Input_Detect channel.

The Data_Retriver layer (data_retriver) is the feature extraction layer + correlation calculation and is responsible for data processing, identifying sensor groups with maximum correlation, and predicting correlation errors. It is a recurrent layer with an attention mechanism for extracting significant temporal correlations. It identifies sensor groups with the highest correlation based on Input_Train data, calculates the correlation from Input_Detect data, and predicts correlation errors.

The Recorder layer (export_jpeg) is the output layer that finalizes the algorithm by saving the correlation analysis results in the diagram.

Recurrent layers (LSTM/GRU) process sequential data, such as the sensor telemetry time series. LSTM and GRU can retain important information about previous states in memory, allowing them to predict future values more accurately, detect dependencies in the data, such as correlations, and predict errors. These layer’s main advantage is their ability to address the vanishing or exploding gradient problem, which often occurs in standard RNNs, especially when working with long sequences.

The Data_Retriver module employs an attention mechanism that focuses on the essential parts of the input data, ignoring less significant ones. It is critical for correlation analysis tasks, as it is necessary to identify key dependencies between sensors in large datasets. The attention mechanism helps the model detect key time points and sensors with high correlations, contributing to more accurate predictions and error localization.

In the Sensor_Fail_Clean and Sensor_Fail_Norm modules, adaptive discretization and quantization allow the data processing parameters to be dynamically adjusted according to their properties. This architecture is essential for preprocessing data before passing them to the recurrent layers.

The Data_Retriver module also implements mechanisms for feature extraction, such as correlation calculations and identifying sensor groups with the highest correlation. This part is responsible for detecting significant relations in the data, which is a critical task in telemetry analysis.

A training algorithm for the proposed recurrent neural network (RNN) was developed based on LSTM or GRU. It includes time-dependent regularization, adaptive gradient correction, and an optimized method that combines stochastic gradient descent (SGD) and RMSProp properties [34,35]. This algorithm considers temporal dependencies and ensures more accurate weight updates, accelerating convergence and enhancing resistance to vanishing gradient problems.

The target function minimizes the prediction error for temporal sequential data *x*^(*t*)^. It is assumed that *y*^(*t*)^ represents the valid values, while ${\hat{y}}^{\left(t\right)}$ represents the model predictions. The loss function defines the error at time *t*:

(17) $$L^{\left(t\right)}=\frac{1}{2}\cdot {\left(y^{\left(t\right)}-{\hat{y}}^{\left(t\right)}\right)}^{2}.$$

The complete loss function for the entire sequence is expressed as follows:

(18) $$L=\sum_{t=1}^{T}L^{\left(t\right)}=\frac{1}{2}\cdot \sum_{t=1}^{T}{\left(y^{\left(t\right)}-{\hat{y}}^{\left(t\right)}\right)}^{2}.$$

In traditional RNNs, gradient issues are often caused by excessively large or small weight values. Time-dependent regularization was introduced to address this, which adaptively adjusts the regularization coefficient based on time *t* during training. L2 regularization with parameter *λ*^(*t*)^ is applied as follows:

(19) $$\lambda^{\left(t\right)}=\lambda_{0}\cdot \frac{1}{1+\alpha \cdot t},$$

where *λ*_0_ is the initial regularization coefficient, and *α* is the regularization decay rate. It reduces the regularization effect in later training stages when network weights stabilize. To counter vanishing gradients, gradient correction is introduced based on the predicted gradient amplitude ${\hat{g}}_{t}$ and the actual value *g_t_*. If the difference between the predicted and actual gradient is too significant, the following correction is applied:

(20) $$g_{t}^{new}=g_{t}\cdot \frac{{\hat{g}}_{t}}{g_{t}+\epsilon },$$

where *ϵ* is a small positive number to prevent the division by zero, allowing the network to avoid abrupt gradient changes and improving training stability.

An optimization method combining RMSProp with stochastic gradient descent (SGD) is employed for more efficient weight updates. It helps account for gradient moments and accelerates convergence during training. The weight update uses an expression with an adaptive training rate as follows:

(21) $$\theta_{t+1}=\theta_{t}-\eta_{t}\cdot \frac{g_{t}}{\sqrt{E{\left[g^{2}\right]}_{t}+\epsilon }},$$

where *g_t_* is the current gradient value, $E{\left[g^{2}\right]}_{t}$ is the exponentially weighted average squared gradient, *ϵ* is a small positive number to prevent division by zero, and *η_t_* is the adaptive training rate, defined as follows:

(22) $$\eta_{t+1}=\frac{\eta_{0}}{1+\beta \cdot t},$$

where *η*_0_ represents the initial training rate, *β* denotes the training rate decay parameter, and *t* indicates the current iteration number.

The update for the exponentially weighted average squared gradient is performed as follows:

(23) $$E{\left[g^{2}\right]}_{t}=\beta \cdot E{\left[g^{2}\right]}_{t-1}+\left(1-\beta \right)\cdot g_{t}^{2}.$$

The training algorithm employs an attention mechanism that assists the model in focusing on significant temporal moments. Attention is calculated as follows:

(24) $$a_{t}=softmax\left(\frac{h_{t}\cdot W_{a}\cdot h_{s}}{\sqrt{d}}\right),$$

where *h_t_* represents hidden states at time *t*, *W_a_* denotes the trainable attention matrix, *h_s_* signifies hidden states from previous time steps, and *d* indicates the hidden state dimensionality. The attention mechanism assists in highlighting the most crucial time steps for the current moment in training, enhancing the model’s ability to handle long sequences.

The developed algorithm outperforms traditional error backpropagation methods due to an adaptive training rate that automatically adjusts based on task complexity, thereby accelerating convergence and avoiding local minima. The gradient correction mechanism prevents abrupt weight changes, enhancing training stability. Control over the mean squared gradient allows for more precise weight updates, while the attention mechanism improves performance with a long time series by focusing on significant moments. These enhancements expedite convergence, reduce computational costs, and increase resilience against vanishing gradients.

## 3. Case Study

### 3.1. Results from the Preliminary Processing of Input Data and Testing Proposed Hypotheses

The research’s experimental parameters include the initial learning rate (*η*_0_ = 0.01) chosen to ensure the model’s smooth convergence in training and minimize weight oscillations in early stages, and the training rate decay parameter (*β* = 0.9) allows the training rate to undergo adaptive reduction as the model stabilizes, reducing the overfitting risk. The hidden state dimension (*d* = 128) ensures complex time-efficient processing dependencies with reasonable computational load. Time dependence regularization, including the initial regularization coefficient (*λ*_0_ = 0.01) and the decay coefficient (*α*), reduces the overfitting and improves the model convergence effect in the late stages. A hybrid method combining SGD and RMSProp is used to optimize the gradient step, which contributes to more robust and faster convergence, especially in the presence of extended data sequences. The correlation threshold (*ϵ* = 0.025) is empirically determined based on helicopter TE characteristics to effectively identify faults while minimizing false alarms. These parameters ensure the model’s high accuracy and robustness under noisy and complex time dependencies, which is confirmed by the experimental results.

**Figure 3:**
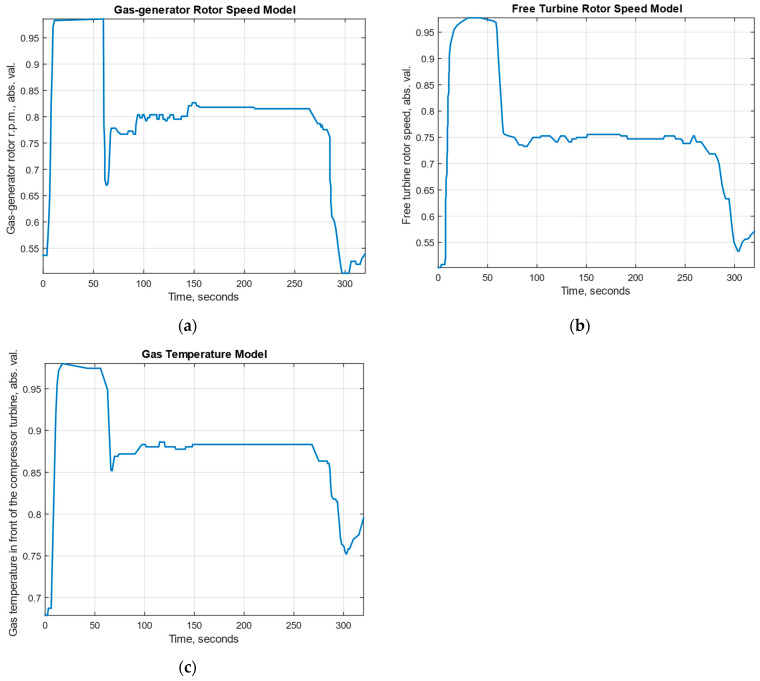
Time series of the TV3-117 turboshaft engine parameter dynamics using digitized oscillograms, where (**a**) is the gas-generator rotor r.p.m.; (**b**) is the free turbine rotor speed; and (**c**) is the gas temperature in front of the compressor turbine.

To conduct the computational experiment, data were collected during TV3-117 turboshaft engine (TE) flight tests aboard the Mi-8MTV helicopter using an onboard control system (recording occurred over a 320-s interval from an actual flight with a 1.0-s sampling period [36]). It is essential to mention that the flight data were acquired following an official request from the author’s team to the Ministry of Internal Affairs of Ukraine as part of the research project titled “Theoretical and applied aspects of the development of the aviation sphere”, which is state-registered in Ukraine under No. 0123U104884. Using the obtained data, a time series diagram for the parameters *n_TC_*, *n_FT_*, and $T_{G}^{*}$ (recorded onboard the helicopter through sensors D-2M, D-1M, and 14 dual thermocouples T-101, respectively [28]) is reconstructed (Figure 3). The TV3-117 TE parameter dynamics reflect the time series shape complexity (Figure 3), and the curves indicate the necessity to consider the parameter values and accumulate information in the model’s memory.

In Figure 3, an increase in parameters is observed in the interval from 21 to 62 s, approximately 1.5 to 1.8 times, which is associated with the engine’s transient operating mode. As mentioned in the introduction, about 85% of the time, the engine operates in steady modes and operates around 15% of the time in transient modes.

Time series are extracted from flight data by extracting successive values of the recorded parameters (*n_TC_*, *n_FT_*, and $T_{G}^{*}$) within each time interval. These data represent discrete measurements that can be interpreted as the time series recording parameter changes over the flight dynamics. Time series are normalized using the *z*-transformation method, where, for the *x_t_* series, each value is transformed according to the following expression: $z_{t}=\frac{x_{t}-\mu }{\sigma_{t}}$, where *x_t_* is the time series value at time *t*, *μ* is the series’ mean value, and *σ* is the series’ standard deviation. This method transforms the data so that their distribution has zero mean and unit variance, which helps to eliminate the scale differences between the influence of different parameters and further improves the analysis efficiency and application of machine learning models.

Thus, according to [36,37,38], 256 values for *n_TC_*, *n_FT_*, and $T_{G}^{*}$ were selected (Figure 3, Table 1).

This size of the training dataset is justified as it provides sufficient coverage of possible variations in helicopter TE parameters, allowing the neural network to train effectively and accurately control engine sensor operations in actual flight conditions. Additionally, a sample of 256 values is adequate to meet the conditions for normal distribution, which is critical for the training dataset’s statistical significance and reliability when applying a neural network for monitoring helicopter TE sensors.

**Figure 4:**
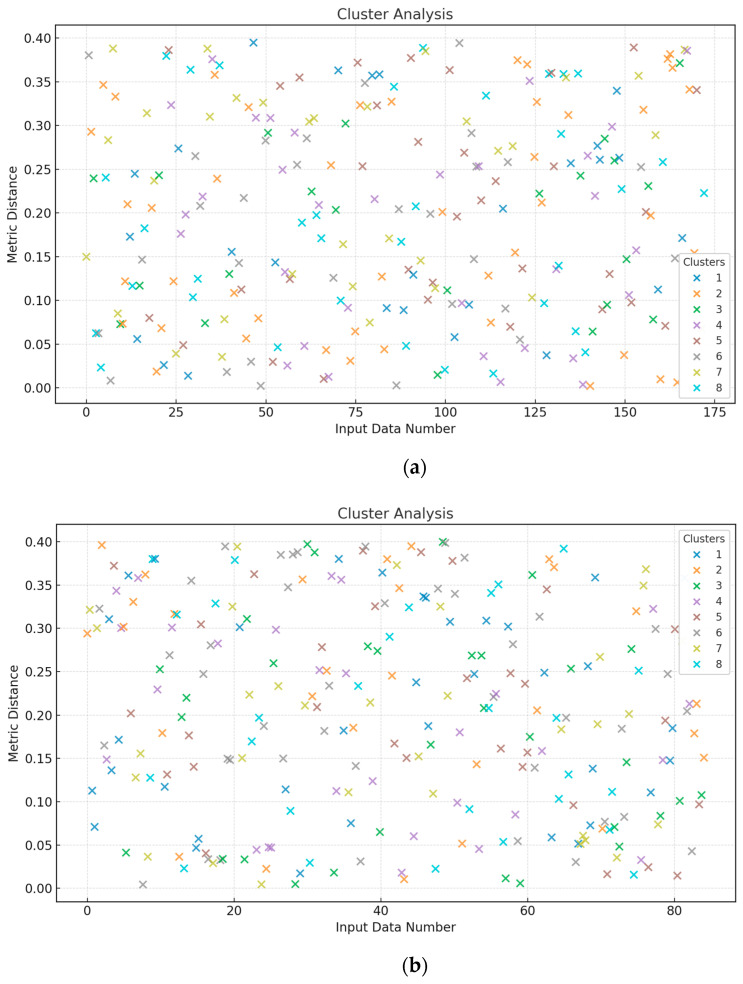
Cluster analysis results: (**a**) training dataset; (**b**) test dataset.

To verify the training dataset’s homogeneity, as outlined in [39,40], a value of *χ*^2^ = 24.915 was calculated, which is less than the critical value $\chi_{critical}^{2}$ = 27.683 with degrees of freedom *df* = 13 and a significance level of *α* = 0.01, indicating homogeneity based on the Fisher–Pearson criterion. The training dataset (*N* = 256) was split into two subsets of 128 elements each. According to [41,42], the Fisher–Snedecor test yielded a value of *F* = 5.565, which is below the critical value *F_critical_* = 5.74, further confirming homogeneity under the Fisher–Snedecor criterion. A cluster analysis was conducted to assess the representativeness of the training and test sets as per [43,44,45]. Based on the data presented in Table 1, the training and test sets were split in a 2:1 ratio (172 and 84 elements). Cluster analysis identified eight classes, confirming the representativeness of both the training and test sets (Figure 4). The optimal sizes are as follows: the training set has 256 elements, the validation set has 172, and the test set has 84.

**Figure 5:**
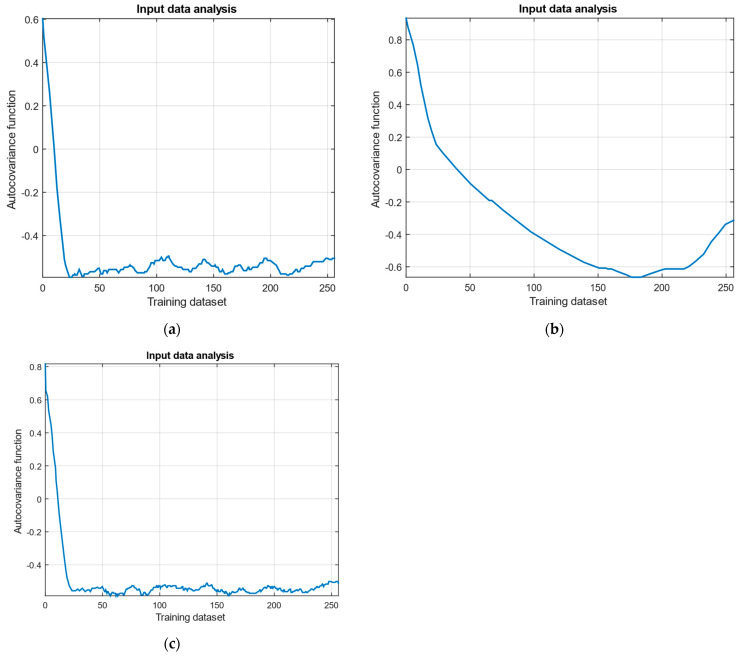
The autocovariance function diagram: (**a**) the gas-generator rotor r.p.m.; (**b**) the free turbine rotor speed; (**c**) the gas temperature in front of the compressor turbine.

To justify the process ergodicity, the Slutsky condition was tested, which states that the autocovariance function of an ergodic process should approach zero as the lag increases [46,47]. The research results confirm the hypothesis regarding the observed processes’ ergodicity. The analysis diagrams for the parameters *n_TC_*, *n_FT_*, and $T_{G}^{*}$ are shown in Figure 5.

### 3.2. Results of Hypotheses 1 and 2 Confirmation

To confirm Hypothesis 1, a computational experiment was conducted to identify interconnected sets of sensors based on telemetry file data, with the results presented in Table 2.

The correlation coefficient values of 0.876 for two sensors (*n_TC_* and *n_FT_*) and 0.913 for three sensors (*n_TC_*, *n_FT_* and $T_{G}^{*}$) confirm a strong and stable correlation among these parameters, suggesting that adequately functioning sensors exhibit consistent interdependencies due to the helicopter TE’s inherent design characteristics. The moderate correlation coefficient of 0.754 for five sensors (*n_TC_*, *n_FT_*, $T_{G}^{*}$, *T_oil_* and *P_oil_*) and 0.735 for six sensors (*n_TC_*, *n_FT_*, $T_{G}^{*}$, *T_oil_*, *P_oil_* and *n_rs_*) demonstrates a slight reduction in correlation as more sensors are added. However, the overall trend supports the hypothesis that functioning sensors are structurally designed to correlate under operational conditions. The correlation analysis further validates the hypothesis by showing that sensor readings maintain robust relations, even when subjected to varying operational parameters, reinforcing the sensor’s performance reliability and predictability in helicopter TE systems. A decrease in correlation coefficients by an average of 23.7% was detected when noise was introduced into sensor readings, indicating their sensitivity to external influences. The results also confirmed the system’s response to simulated device failure, reflecting altered dynamics in correlations between parameters.

**Figure 6:**
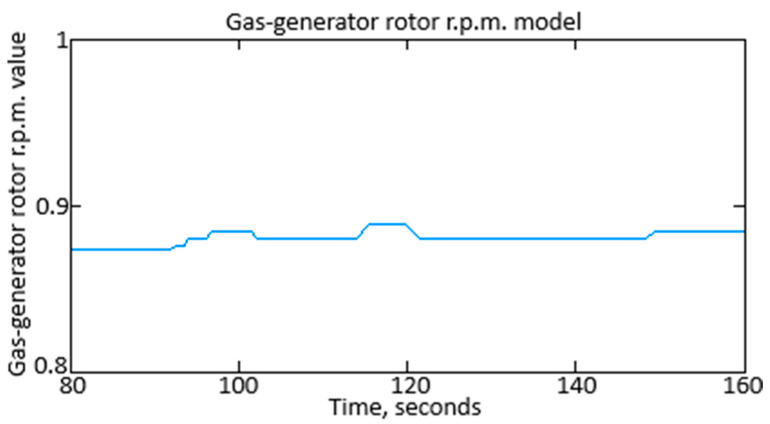
The diagram fragment shows readings from the gas-generator rotor r.p.m.

To confirm Hypothesis 2, the fault detection issue was considered using the *n_TC_* parameter as an example. An *n_TC_* recording was generated and linked to a time scale in the measurement channel. The measurements formed in this manner created an array that also contained false information regarding changes in the *n_TC_* due to the noise influence. Next, the actual value was calculated using an approximating polynomial that considered the helicopter TE’s fundamental characteristics while “ignoring” the presence of a sensor’s fault caused by interference. The fault assumption is based on the fact that sensor readings may not comply with the physical laws governing the helicopter TE’s operation. For instance, a sudden change in the range sensor reading followed by a return to the initial position indicates noise presence or a sensor fault, as this does not align with the helicopter TE’s operational dynamics. Such readings must be recognized and corrected by filtering out the noise. Figure 6 illustrates a short-term disturbance effect on the range characteristic, allowing for the fault’s presence and visual observation.

**Figure 7:**
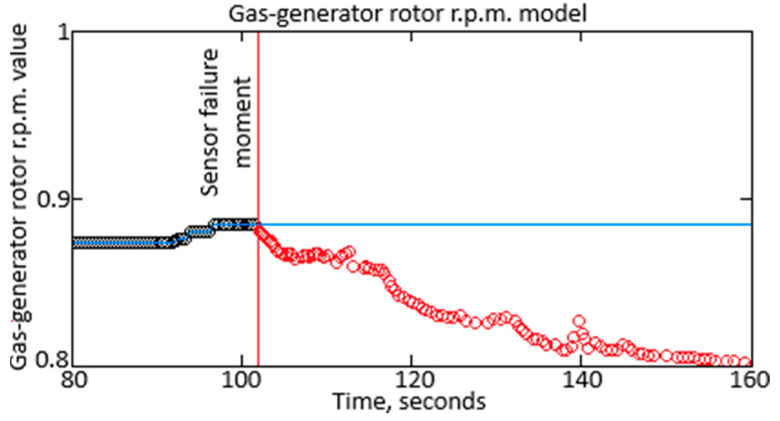
The diagram fragment shows readings from the gas-generator rotor r.p.m. considering the sensor malfunctions.

As the experimental research result, Δ = 0.025 was accepted. In Figure 7, the sensor readings are shown in black; the data restored by the polynomial are presented in light blue; and the moments when the measured value deviations exceeded the allowable threshold are marked in red. The sensor failure occurred at 102 s, after which the sensor began providing inadequate readings.

**Figure 8:**
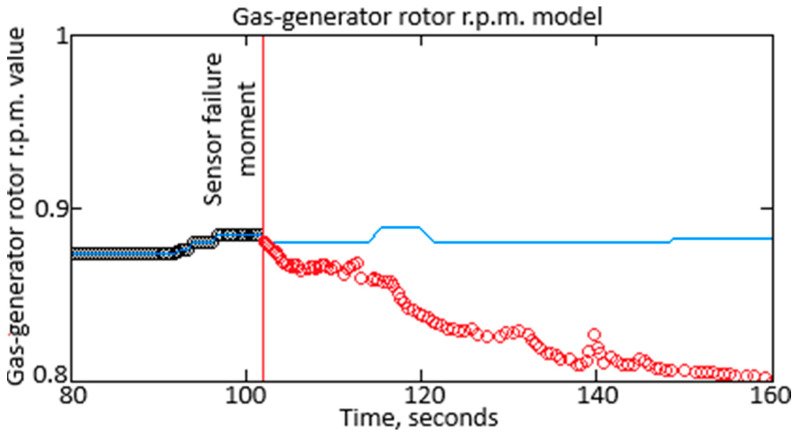
The diagram fragment shows readings from the gas-generator rotor r.p.m. considering the sensor readings restored without failures.

The point weights are redistributed in the third step, and the polynomial is recalculated using the developed neural network (Figure 2). This approach enhances the accuracy of sensor reading’s interpolation. The neural network’s output is shown in Figure 8, where the *n_TC_* sensor’s actual readings are marked in black, and the restored values, based on the analysis of readings and fault detection, are displayed in blue.

### 3.3. Results from Solving the Fault Detection Problem Using the Correlation Dependency Analysis Method

**Figure 9:**
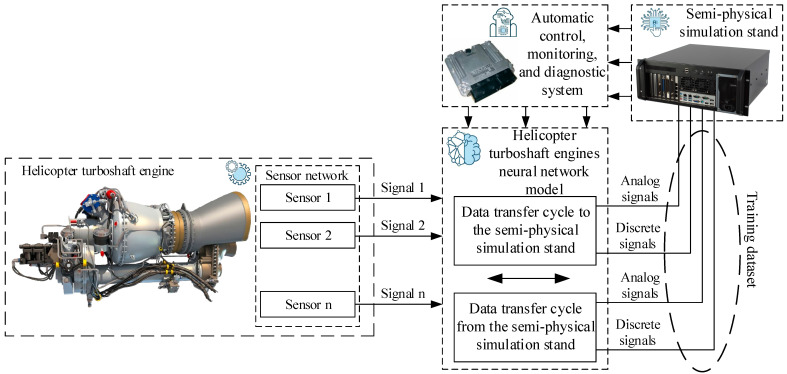
Structure for integrating the helicopter turboshaft engine model with the developed semi-physical simulation stand.

A computational experiment was conducted using a developed semi-physical simulation stand (SPSS) (Figure 9) designed to simulate helicopter TE parameters in real time and model their operating modes across various altitudes and flight speeds. The SPSS also interacts with upper-level systems via data channels, tests the automatic control, monitoring, and diagnostic system (ACMDS), and addresses other tasks [48,49,50]. Figure 9 illustrates the interaction between the neural network and the SPSS. The helicopter TE model operates in two-time cycles, each executed on a separate CPU core (for the computational experiment in this study, an AMD Ryzen 5 5600 processor with 3.5 GHz, six cores, and 12 threads, Germany, Dresden, was used).

**Figure 10:**
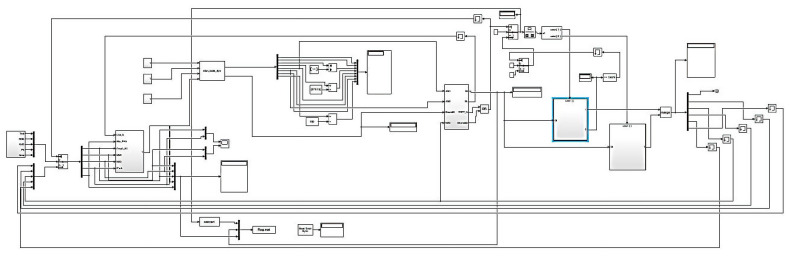
Overview illustrating the interaction between the proposed neural network and the developed semi-physical simulation stand within the Matlab Simulink R2014b environment.

The high-priority cycle processes data from the SPSS and runs the neural network, while the lower-priority cycle sends the results back to the SPSS, ensuring real-time operation. The SPSS functions within the Matlab Simulink R2014b graphical environment (version 2014b), where data exchange channels, a fuel valve emulator, a visualization system, and other components are implemented (Figure 10). The neural network is integrated into Simulink using built-in tools, facilitating seamless model implementation [50].

The semi-physical modelling stand diagram, developed in MATLAB Simulink R2014b (Figure 10), is a complex system that includes input, computational and output modules. Input signals are formed in data generation blocks that model physical processes or external influences and can also come from sensors or real devices. These signals are transmitted through multiplexers, which combine them into a vector for processing. The diagram’s central part contains computational modules that include the control object’s models, calculation and filtering algorithms, and control and feedback subsystems. The system’s dynamics are modelled, taking into account the object’s parameters, and the feedback blocks are corrected based on the output parameter’s deviations from the specified values. The diagram also contains modules for storing intermediate results and constant parameters, presented in the memory blocks and constants formed, which allow the system to be adapted to changing conditions or simulation scenarios. Logical switches and control blocks enable operating modes and switch signal processing trajectories to be set. The output data are sent to visualization units such as graphical interfaces or data logging tools and can also be transmitted to real devices for control or analysis.

**Figure 11:**
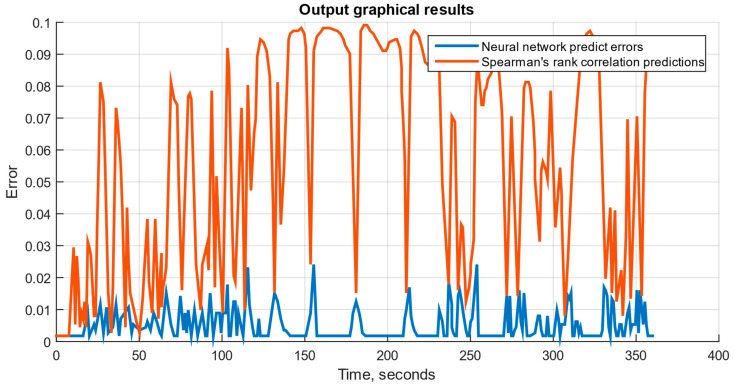
The diagram for the set *n_TC_* and *n_FT_*.

**Figure 12:**
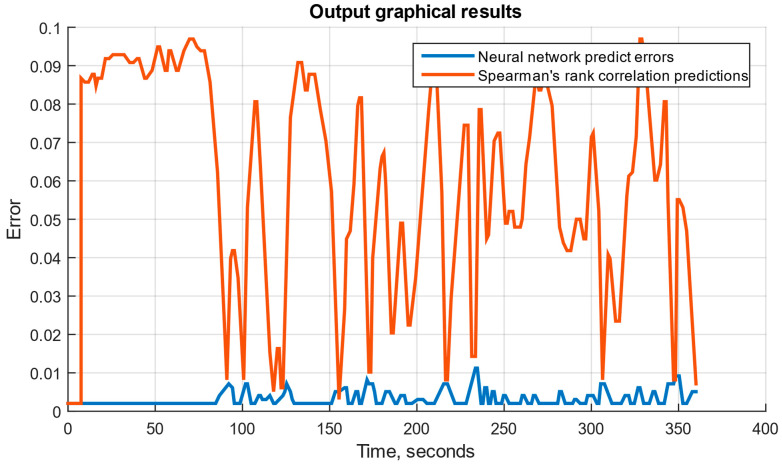
The diagram for the set with *n_TC_*, *n_FT_*, and $T_{G}^{*}$.

**Figure 13:**
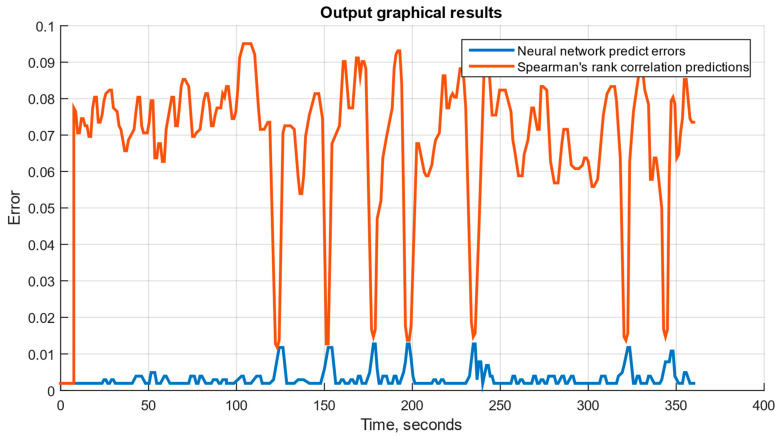
The diagram for the set with *n_TC_*, *n_FT_*, $T_{G}^{*}$, *T_oil_*, *P_oil_*, and $n_{rs}$.

**Figure 14:**
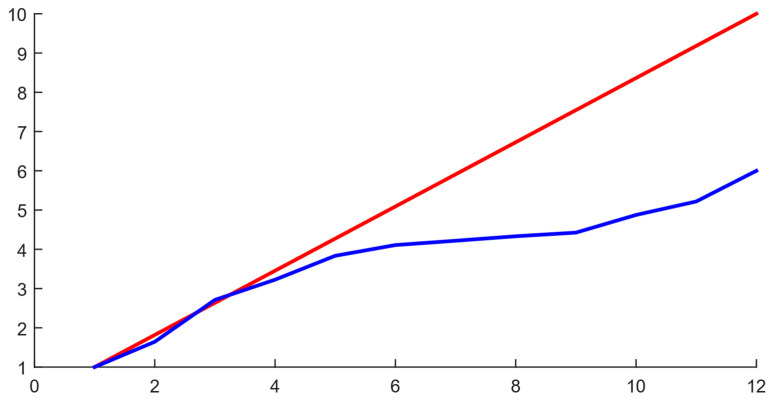
The diagram illustrating the acceleration in computation as the number of threads increases: (red curve) the linear regression model; (blue curve) the obtained values on the AMD Ryzen 5 5600 3.5 GHz processor, 6 cores, 12 threads.

If the neural network module operates correctly, three graphical files can be generated: a diagram for the set *n_TC_* and *n_FT_*; a diagram for the set *n_TC_*, *n_FT_*, and $T_{G}^{*}$; and a diagram for the set *n_TC_*, *n_FT_*, $T_{G}^{*}$, *T_oil_*, *P_oil_* and *n_rs_*. These diagrams are shown in Figure 11, Figure 12 and Figure 13, where the neural network prediction errors are represented in blue, and Spearman’s rank correlation [51,52] predictions for the sensor set are shown in red. The testing results are presented in Figure 14 and Table 3.

Table 3 demonstrates a significant reduction in computation time as the number of cores and threads increases, with the relative time improving up to six times when using all 12 threads. However, the performance gains diminish as the core and thread count increase beyond eight, suggesting a levelling effect in efficiency improvements. The results enable research in the time series value approximation field, opening opportunities for more accurate dynamic process modelling and analysis.

### 3.4. Results from Time Series Value Approximation

**Figure 15:**
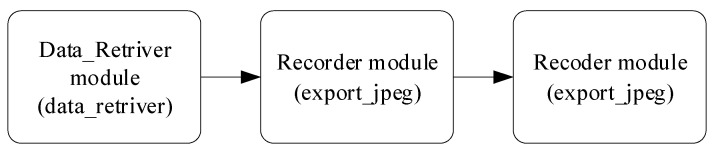
The proposed algorithm’s block diagram for solving the approximating the time series values task.

In the computational experiment’s next stage, the approximation task of time series values under the sensor failure conditions is addressed (Figure 15). The input data consist of readings from six sensors and their corresponding time values (Table 4). The input data processing algorithm is represented as follows:

- Step 1. The Data_Retriever module (data_retriver) sends telemetry data to the Sensor_Fail module (sensor_fail) via the Input channel;
- Step 2. The Sensor_Fail module (sensor_fail) processes the telemetry data and transfers an image showing both raw and smoothed telemetry data to the Recorder module (export_jpeg);
- Step 3. The Recorder module (export_jpeg) saves the generated images.

**Figure 16:**
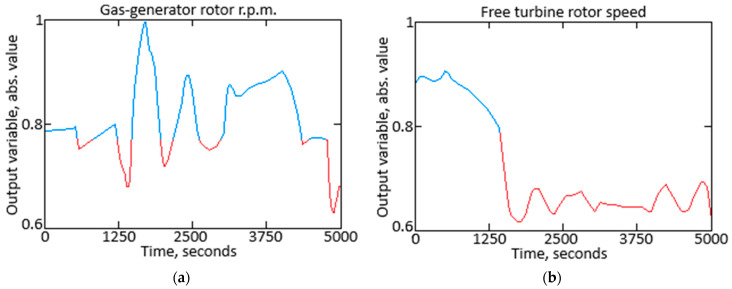

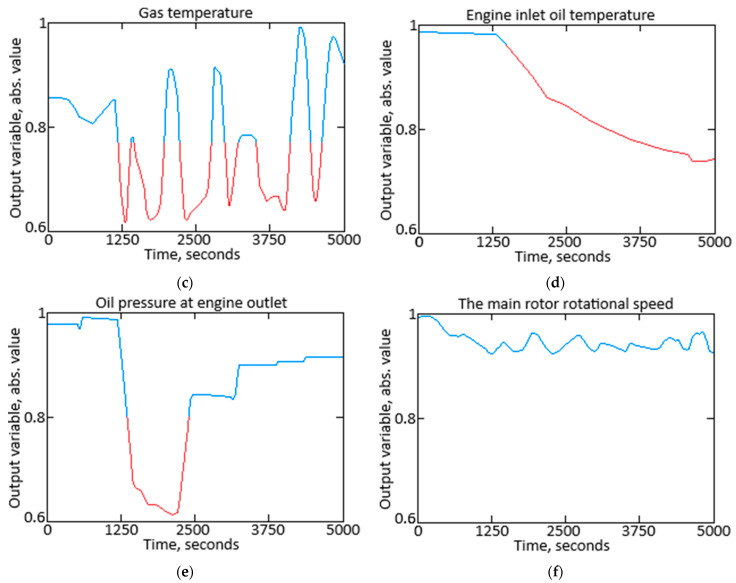
The results of approximating time series values under the sensor failure conditions task: (**a**) Gas generator rotor speed sensor; (**b**) Free turbine rotor speed sensor; (**c**) Gas temperature in front of the compressor turbine sensor; (**d**) Engine inlet oil temperature sensor; (**e**) oil pressure at engine outlet sensor; (**f**) The main rotor rotational speed sensor.

Figure 16 shows the detected sensor failures (visually undetectable). The implementation of the Sensor_Fail module does not support the processing of parallel input data at this stage. During SPSS testing (Figure 10) using a single unit, the results presented in Table 5 were obtained.

### 3.5. Results of Neural Network Quality Assessment

An evaluation of the developed neural network (Figure 2) performance was conducted using key quality metrics such as accuracy, loss, precision, recall, the F1-metric [53,54,55], and AUC-ROC [56,57]. These metrics allow the model to undergo a comprehensive assessment to make accurate predictions, minimize errors, correctly identify relevant instances, and maintain a balance between precision and recall. The F1-metric offers insight into the precision and recall harmonic mean, while AUC-ROC measures the model’s capability to distinguish between classes across different thresholds, ensuring robustness in various operational scenarios. These metrics are calculated according to the following expressions [53,55,56]:

(25) $$\begin{array}{c} Accuracy=\frac{1}{N}\cdot \sum_{i=1}^{N}1\left(y_{i}={\hat{y}}_{i}\right),\;Precision=\frac{TP}{TP+FP},\;Recall=\frac{TP}{TP+FN},\\ F1-score=\frac{2\cdot Precision\cdot Recall}{Precision+Recall},\;AUC-ROC=\int_{0}^{1}TPR\cdot \left({FPR}^{-1}\left(t\right)\right)dt. \end{array}$$

In this context, *y_i_* represents the actual label for the *i*-th instance, while ${\hat{y}}_{i}$ stands for the predicted label produced by the model for that same instance. *N* indicates the total count of examples in the dataset (whether for the training or validation dataset), and the indicator function $1\left(y_{i}={\hat{y}}_{i}\right)$ returns a value of 1 if the true and predicted labels are equal and returns a value of 0 otherwise. In the context of fault detection and sensor data restoration, *TP* (True Positive) refers to cases when a fault was correctly identified, and the sensor recorded erroneous values. *TN* (True Negative) signifies instances where the system correctly recognized the absence of faults, and the sensor readings were accurate. *FP* (False Positive) describes situations where the system mistakenly detected a fault, though the sensor data were correct. *FN* (False Negative) represents cases where a fault was present, but the system failed to detect it. $TPR=\frac{TP}{TP+FN}$ (True Positive Rate) measures the proportion of correctly identified faults from all actual faults, while $FPR=\frac{FP}{FP+TN}$ (False Positive Rate) indicates the false alarm proportion when the system wrongly identified faults.

**Figure 17:**
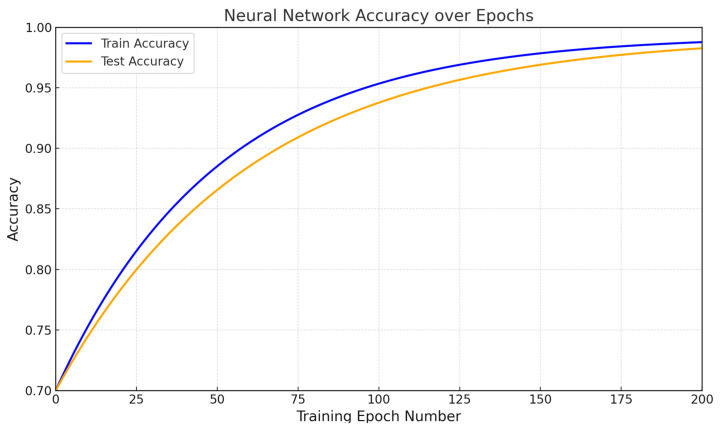
Accuracy metric diagram.

**Figure 18:**
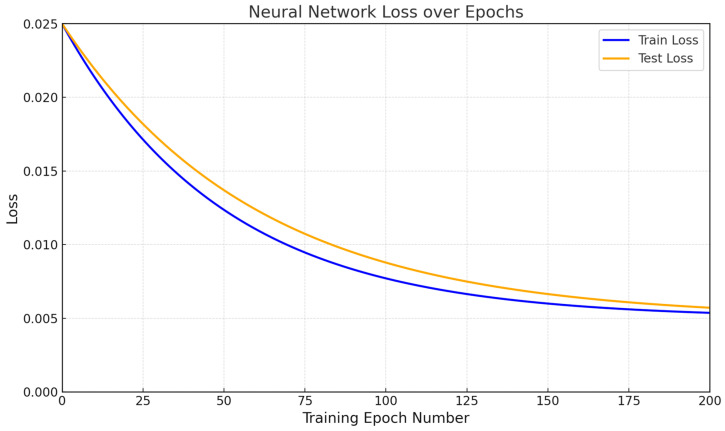
Loss metric diagram.

Figure 17 and Figure 18 show accuracy and loss metrics diagrams. The developed neural network (Figure 2) accuracy metric reaches 99.327% after 200 training epochs to solve the fault detection in sensor-recorded values during the restoring task. This high performance demonstrates the model’s effectiveness in identifying anomalies and restoring correct data under varying conditions. The loss of the developed neural network (Figure 2) decreases from 2.5 to 0.5% after 200 training epochs to solve the fault detection in sensor-recorded values during the restoring task. This loss reduction reflects the network’s improved accuracy and stability in handling sensor data anomalies.

The developed neural network achieved a precision score of 0.987, a recall score of 1.0, and an F1-score of 0.993 in the sensor-recorded values during the fault detection and restoring task. These metrics indicate high accuracy and reliability in identifying faults while maintaining zero False Negatives. The F1-score demonstrates the model’s balanced performance in precision and recall terms.

**Figure 19:**
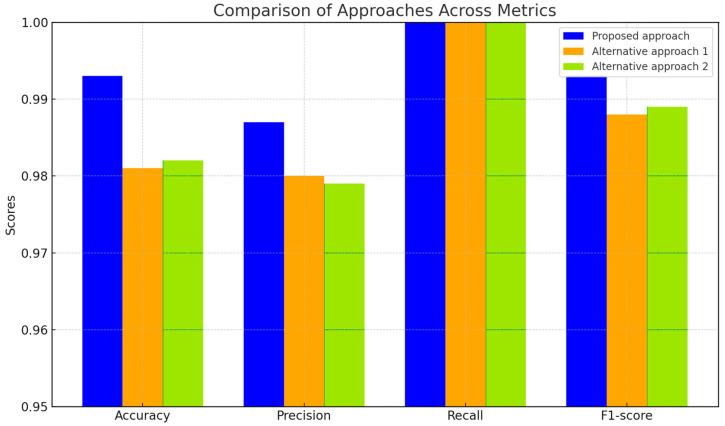
Comparative analysis results.

**Figure 20:**
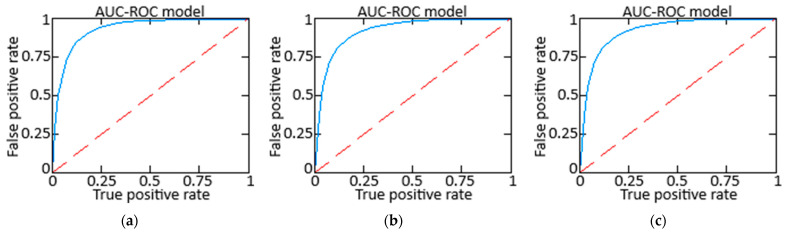
The AUC-ROC curve: (**a**) the proposed approach; (**b**) Alternative approach 1; and (**c**) Alternative approach 2.

The obtained results were compared (Table 6 and Table 7, Figure 19 and Figure 20) with two alternative approaches: approach 1 is a method for restoring lost information when sensors fail using an auto-associative neural network (autoencoder) [37], and approach 2 is a neural network system for predicting anomalous data based on the SARIMAX model with LSTM blocks [58].

The comparative results reveal that the proposed approach outperforms both alternative methods in terms of accuracy (0.993 vs. 0.981 and 0.982), precision (0.987 vs. 0.980 and 0.979), and F1-score (0.993 vs. 0.988 and 0.989). Notably, all approaches achieved the same recall score of 1.0, indicating that they effectively detected all actual faults. However, the superior accuracy and precision of the proposed method underscore its potential for enhanced reliability in fault detection and the restoration of values in helicopter TE sensor systems.

The monitoring system successfully detected 99 cases of real sensor failures, which indicates its ability to record significant deviations in readings. These could be failures such as mechanical damage to the rotors (D-2M and D-1M sensors), electromagnetic interference or component wear, and incorrect temperature measurements due to damage to the thermocouple (T-101 sensor). However, 13 real failures remained unnoticed. These cases are likely due to minor parameter deviations or complex defects that the system erroneously classified as normal. At the same time, the system gave 289 False Positives, erroneously classifying normal readings as failures. This may be due to their high sensitivity to noise, measurement errors or inadequate classification thresholds. This situation leads to an increase in the number of false alarms.

To improve the system’s performance, it is necessary to optimize the data processing algorithms, including noise filtering, threshold adjustment, and refinement of the model to detect subtle faults. It is also recommended that individual fault types for each sensor are analyzed to understand which faults the system ignores or misclassifies. It will improve the classification accuracy and reduce both False Positives and False Negatives, which is critical to ensuring the helicopter TE’s reliability.

The comparative analysis based on AUC-ROC metrics (Figure 20) demonstrates that the proposed approach achieves a superior AUC-ROC score of 0.823, outperforming both alternative methods, which scored 0.818 each. This improvement is accompanied by an actual positive rate of 0.822, indicating a robust capability to identify actual faults while maintaining a low False Positive rate of 0.0111. The lower number of False Negatives (13) further highlights the proposed method’s effectiveness in minimizing missed detections compared to alternative approaches.

**Figure 21:**
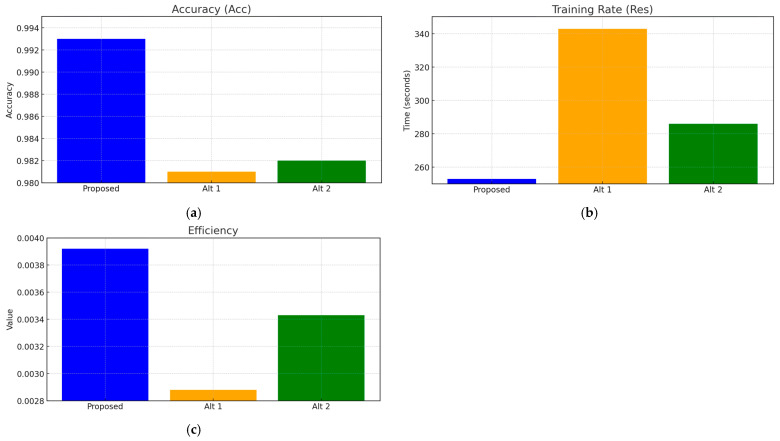
Comparative analysis results: (**a**) accuracy metric; (**b**) training rate metric; and (**c**) efficiency metric.

To evaluate the proposed approach’s effectiveness in efficiency terms, this research utilizes the *Efficiency* metric, which incorporates both prediction accuracy and the resources consumed during training and/or inference. This metric can represent the relationship between accuracy and resource usage (such as computational power or time). Here, *Acc*_1_, *Acc*_2_, and *Acc*_3_ signify the three neural network accuracies under comparison (the proposed neural network (Figure 2), the auto-associative neural network (autoencoder) [37], and the SARIMAX model with LSTM blocks [58]), while *Res*_1_, *Res*_2_, and *Res*_3_ represent the resources employed (in this investigation, the training time parameter was adopted, as both networks were evaluated under an identical memory capacity of 32 GB DDR-4). The resource efficiency (Table 8 and Figure 21) is then calculated using the following expression:

(26) $${Efficiency}_{1}=\frac{{Acc}_{1}}{{Res}_{1}},{\;\;\;Efficiency}_{2}=\frac{{Acc}_{2}}{{Res}_{2}},\;{\;\;\;Efficiency}_{3}=\frac{{Acc}_{3}}{{Res}_{3}}.$$

The comparison results indicate that the proposed approach achieves an accuracy of 0.993 while maintaining a training time of 4 min and 13 s, resulting in a resource efficiency metric of 0.00392. In contrast, Alternative Approach 1 demonstrates an accuracy of 0.981 with a longer training duration of 5 min and 43 s, yielding an efficiency value of 0.00288. Similarly, Alternative Approach 2 achieves an accuracy of 0.982 with a training time of 4 min and 46 s, resulting in a resource efficiency metric of 0.00343.

All three approaches demonstrate optimal and efficient performance for implementation onboard a helicopter. However, the proposed approach exhibits a slight accuracy and resource efficiency advantage, making it the preferred choice for real-time applications in this context.

## 4. Discussion

This article presents a neural network system for monitoring helicopter TE sensors (see Figure 1). The developed system includes the following modules: the clean_parser module sends telemetry data for smoothing to the Sensor_Fail_Clean module via the Input channel; the norm_parser module transfers data for normalization to the Sensor_Fail_Norm module through the Input channel; the Sensor_Fail_Clean module smooths the data, performs adaptive discretization and quantization, and then passes it to the Data_Retriever module through the Input_Train channel; and the Sensor_Fail_Norm module normalizes the data and sends it to the same module via the Input_Detect channel. The Data_Retriever module identifies sensor groups with maximum correlation using data from Input_Train, calculates correlations from Input_Detect, and detects predicted errors. The Recorder module saves the diagrams of the results obtained through the Input channel. This system is implemented as a hybrid LSTM/GRU neural network (see Figure 2). The recurrent layers (LSTM/GRU) process sequential data, such as telemetry time series, retaining key information about previous states for more accurate prediction and dependency identification, including correlations and forecast errors. Their advantage is that they overcome the vanishing or exploding gradient problem, especially when working with long sequences. The Data_Retriever module uses an attention mechanism to focus on significant parts of the data, which is crucial for correlation analysis, highlighting critical dependencies between sensors. The Sensor_Fail_Clean and Sensor_Fail_Norm modules apply adaptive discretization and quantization for dynamic processing parameter adjustments, improving data quality before feeding this into the recurrent layers. The Data_Retriever model also performs correlation calculations and identifies sensor groups with the highest correlation, which is essential for telemetry analysis.

An algorithm for training the proposed neural network based on a hybrid LSTM/GRU architecture (see Figure 2) has been developed, incorporating temporal regularization, adaptive gradient correction, and a combined optimization method that integrates stochastic gradient descent (SGD) with RMSProp (17)–(24). This algorithm accounts for temporal dependencies in the data, enabling more accurate weight updates, which accelerate convergence and enhance resistance to vanishing gradients (17)–(24).

Based on the helicopter TE and its structural features, the computational experiment focuses on solving sensor system monitoring tasks under interference conditions by forming and testing hypotheses regarding correlation and data reliability. The monitoring process considers Hypotheses 1 and 2 (1)–(5).

The developed methods for testing Hypotheses 1 and 2 include a comprehensive approach to analyzing sensor readings for detecting correlations and identifying failures. The first method (6)–(11) focuses on testing the hypothesis regarding the pairwise presence and triple correlations between readings from various helicopter TE sensors. It involves adaptive discretization, signal quantization, and analysis using discrete wavelet transform, which helps reveal hidden patterns and anomalies. Correlation coefficients are also calculated using sliding windows to find optimal sensor combinations with the highest correlation. The second method (12)–(16) targets failure detection in individual sensor readings, such as compressor turbine rotor speed, by approximating actual characteristics with a polynomial. Anomalies are identified based on significant deviations between the measured data and approximated values. It allows interference to be filtered out and measurement accuracy to be improved through polynomial recalibration, considering the weights for each point.

The computational experiment used data recorded during TV3-117 engine flight tests on a Mi-8MTV helicopter through the onboard monitoring system. The recordings were made over 320 s of actual flight, with a sampling interval of 1 s (see Figure 3). The engine parameter dynamics revealed the time series complexity, and the nature of the curves highlighted the need to account for parameters and accumulate information in the model’s memory for accurate analysis. Based on the collected data, a training dataset was created (see Table 1), with its uniformity confirmed by Fisher–Pearson and Fisher–Snedecor criteria and its representativeness validated by cluster analysis (see Figure 4).

To confirm Hypothesis 1, a computational experiment was conducted to identify interrelated sets of sensors based on telemetry data (see Table 2). The experiment revealed the highly correlated sensor’s presence, a reduction in correlation when interference was introduced, and the system’s response to simulated failures. Based on these results, Hypothesis 1 was validated.

To confirm Hypothesis 2, an experiment was conducted to detect failures using the gas-generator rotor r.p.m. parameter as an example (see Figure 6). The measured data, including false changes caused by interference, were analyzed using an approximating polynomial obtained through traditional methods (e.g., least squares method) (see Figure 7) and the proposed neural network (see Figure 8). The polynomial accounts for the helicopter TE’s characteristics and ignores sensor failures. A failure is detected if sensor readings do not match the helicopter TE operation dynamics, such as sharp changes and returns to initial values. The experiment confirmed the method’s effectiveness, applying a threshold value of Δ = 0.025. Data correction was improved by redistributing weights and using the neural network, which increased the accuracy of reading interpolation.

The developed system is implemented as an SPSS (see Figure 9), designed to simulate helicopter TE parameters and model their operating modes across an altitude range and flight speeds. The SPSS was implemented in the Matlab Simulink graphical environment, where data exchange channels, a fuel metering unit simulator, a visualization system, and other components were integrated (see Figure 10). The trained neural network was integrated into Simulink using built-in tools, simplifying the model’s implementation. The system’s performance, using an AMD Ryzen 5 5600 processor with six cores and 12 threads, was confirmed by output graphical files (see Figure 11, Figure 12 and Figure 13). However, performance gains decreased as the number and threads of the core exceeded eight, indicating a levelling effect on efficiency improvement (see Figure 14 and Table 3).

The task of approximating time series values recorded by helicopter TE sensors was solved in this research (see Figure 15). The neural network system from successfully identified segments where the recorded parameter data were anomalous (see Figure 16).

The effectiveness of the developed neural network system was evaluated using traditional quality metrics. The designed neural network (see Figure 2) achieved an accuracy of 99.327% after 200 training epochs for detecting and restoring faults based on sensor data (see Figure 17). This high performance demonstrates the model’s effectiveness in identifying anomalies and restoring correct data under various conditions. The network’s loss decreased from 2.5 to 0.5% (see Figure 18) over the same period, reflecting improved accuracy and stability when processing anomalies in sensor data. The developed neural network showed precision at 0.987, recall at 1.0, and F1-score at 0.993, indicating high accuracy and reliability in identifying faults without False Negatives. The F1-score also highlighted the model’s balanced performance in the precision and recall terms.

The obtained results were compared (see Table 6 and Table 7 and Figure 19) with two alternative approaches: 1 is a method for restoring lost information during sensor failures using an auto-associative neural network (autoencoder), and 2 is a neural network for predicting anomalous data based on the SARIMAX model with LSTM blocks. The comparative analysis showed that the proposed approach outperformed both alternative methods in accuracy (0.993 vs. 0.981 and 0.982), precision (0.987 vs. 0.980 and 0.979), and F1-score (0.993 vs. 0.988 and 0.989). In contrast, all approaches demonstrated the same recall value of 1.0, confirming their ability to detect all actual failures effectively. Analysis based on AUC-ROC metrics (see Figure 20 and Table 7) also revealed that the proposed method achieved an AUC-ROC of 0.823, surpassing both alternative methods with a score of 0.818. At the same time, the valid positive rate stood at 0.822, with a low False Positive rate of 0.0111. The False Negative number (13) highlights the proposed approach’s effectiveness in minimizing misses compared to alternative approaches. An efficiency metric assessed the proposed approach’s effectiveness, considering prediction accuracy and the resources spent during training and/or inference. The comparison results (see Table 8 and Figure 21) indicate that the proposed method achieved an accuracy of 0.993 with a training time of 4 min and 13 s, resulting in a resource efficiency value of 0.00392. In contrast, Alternative Approach 1 showed an accuracy of 0.981 with a longer training time of 5 min and 43 s, yielding an efficiency value of 0.00288, while Alternative Approach 2 achieved an accuracy of 0.982 with a training time of 4 min and 46 s, leading to a resource efficiency metric of 0.00343. All three approaches demonstrated optimal and adequate performance for implementation on board a helicopter; however, the proposed method holds a slight advantage in accuracy and resource efficiency, making it the preferred choice for real-time applications.

The developed experimental complex (see Figure 9 and Figure 10) addresses tasks such as interpolating numerical series from sensor readings using the least squares method, predicting values from numerical series using a hybrid LSTM/GRU neural network architecture (see Figure 2), as well as monitoring and diagnosing helicopter TE sensors. All tasks were tested with consideration for parallel processing on a cluster setup, receiving data for processing at a rate of 256 Kbps. Table 9 presents the results for various tasks performed by the experimental complex based on (see Figure 9 and Figure 10), tested on a node with a performance of 7.0 GFLOPS. The proposed methods for increasing performance involve installing graphics processors for parallel data processing and deploying a high-speed switching network for information exchange between nodes in the cluster system.

The proposed algorithm outperforms other methods by combining advanced approaches aimed at improving the model’s performance and accuracy. Using a hybrid architecture based on LSTM and GRU, it can efficiently process time series data such as telemetry while preserving key information about previous states. It ensures the more accurate detection of dependencies between sensor readings and anomaly detection. By using adaptive optimization methods such as a combination of stochastic gradient descent (SGD) with RMSProp, the proposed algorithm takes into account data temporal dependencies, accelerates convergence, and minimizes the vanishing gradients problem, which is especially important when working with long sequences.

The built-in attention mechanism in the Data_Retriever module improves model performance by focusing on the data’s most significant parts. It allows for the identification of critical dependencies between sensor readings and increases the accuracy of correlation analysis. The Sensor_Fail_Clean and Sensor_Fail_Norm modules provide data preprocessing, including adaptive discretization and quantization, which improves the quality of input data and minimizes the impact of noise and interference. This preliminary data improvement can help reduce errors and enhance model stability.

A comparison with alternative methods such as autoencoders and SARIMAX models showed that the proposed algorithm achieved higher accuracy (0.993 vs. 0.981 and 0.982), with a shorter training time (4 min 13 s vs. 5:43 and 4:46) and better resource efficiency. This was achieved through a balanced training approach that included temporal regularization, adaptive gradient correction, and optimized data processing mechanisms. Such characteristics make the proposed method preferable for real-world applications, especially in settings requiring high reliability and minimal computational costs.

In this research, the developed neural network-based system for monitoring helicopter TE sensors demonstrates significant efficiency in detecting anomalies and restoring data; however, the research results have certain limitations. The model’s accuracy in real-time conditions may vary depending on the quality of input data and the presence of interference, potentially reducing the prediction’s reliability. The adaptive discretization and quantization algorithms used for data processing may be sensitive to changes in operational conditions, requiring additional parameter adjustments. Furthermore, while the applied validation methods show high accuracy, they are limited to the specific conditions under which the experiments were conducted. They may not always directly apply to other engine models or various helicopter types. Implementing this system on processors with limited computational capacity could affect data processing speed, which is critical in dynamic flight conditions, where data acquisition and processing delays may compromise safety levels.

In this research, an efficient neural network-based system for monitoring helicopter TE sensors was developed. Still, several areas for future research could significantly expand its functionality and improve diagnostic accuracy. Integrating deep learning methods, such as convolutional neural networks (CNNs) [59,60,61], should be considered for processing multidimensional data and enhancing anomaly detection under complex interference conditions, which could improve prediction accuracy and system adaptability to different operational scenarios. Incorporating active training mechanisms [62,63,64] and online adaptation [65,66] could allow the system to train from new data in real time, increasing its resilience to changing working conditions and enhancing the model’s generalization ability. The third direction involves using ensemble training methods [67,68] to combine predictions from multiple models, potentially reducing variability and increasing prediction reliability. Moreover, the development of the neural network system (Figure 2) brings further improvements, including the directions presented in Table 10.

In addition, the algorithm’s performance may be degraded under extreme conditions, such as sudden changes in environmental parameters, strong vibrations, or temperature variations, which may increase noise in the data or change the sensor’s characteristic signals. These factors may complicate fault identification and increase the number of False Positives. For example, 289 False Positives, in which normal values were incorrectly classified as faults, indicate the system’s high sensitivity to deviations in the data, including noise or minor parameter fluctuations. This situation may occur due to insufficient data filtering, too-strict trigger thresholds, or incorrectly configured classification criteria. This leads to an increased workload for system operators as a large number of false alarms must be checked, which complicates the operation and reduces the efficiency of the system in real time. Future work should consider the ability to adapt the model to extreme conditions, for example, by expanding the training set with data collected in different operating modes and implementing noise-robustness techniques, such as more complex filters or improved attention mechanisms to focus on critical data features.

Additionally, exploring methods for interpreting and explaining model decisions [69,70,71] appears promising, as this could improve trust in the results and provide operators with valuable recommendations for decision-making based on data analysis. Furthermore, implementing a monitoring system using drones [72] or mobile platforms [73] for automatic data collection in hard-to-reach areas [74] could improve telemetry quality and broaden the system’s applicability in various conditions [75,76]. There are plans to explore the developed system with other equipment and components onboard the helicopter [77,78,79] to create a comprehensive system for controlling operational status and safety [73,74,75,76,77], significantly enhancing reliability [78,79,80,81,82,83] and flight safety [84,85,86,87].

## 5. Conclusions

The developed hybrid neural network model, combining LSTM/GRU architectures, adaptive learning rates, and attention mechanisms, represents an innovative approach in the field of monitoring helicopter turboshaft engine sensors. This combination allows the efficient processing of sequential data and significantly improves the accuracy of anomaly detection, which is especially important for ensuring the helicopter turboshaft engine’s safety and reliability. The use of recurrent layers (LSTM/GRU) provides vital information about previous state preservation, which is critical for time series analysis and identifying dependencies in the data.

The Sensor_Fail_Clean and Sensor_Fail_Norm modules, implementing data-adaptive discretization and quantization, can improve the input data quality transmitted to the neural network, which, in turn, contributes to more accurate prediction and analysis. The effectiveness of the proposed model was confirmed by high accuracy in detecting anomalies and restoring data, with a result of 99.327% accuracy after 200 training epochs, which indicates the system’s stability even when processing abnormal situations.

The developed neural network system for helicopter turboshaft engine sensors demonstrates high accuracy in anomaly detection and data restoration, achieving 99.327% accuracy after 200 training epochs. Network loss decreases from 2.5 to 0.5%, indicating improved stability when processing anomalies.

Developed a training algorithm that includes time regularization and optimization methods (SGD combined with RMSProp), which accelerates the convergence of neural networks, enhances resistance to gradient-vanishing problems, and improves training efficiency and model stability to a certain extent. This is evidenced by the reduction in the training time to 4 min and 13 s with an accuracy of 0.993.

When compared to alternative methods (auto-associative neural network and SARIMAX model with LSTM), the proposed method outperforms across all key metrics: accuracy is 0.993 versus 0.981 and 0.982, precision is 0.987 versus 0.980 and 0.979, and F1-score is 0.993 versus 0.988 and 0.989, confirming its superiority in fault detection and restoration.

Our computational experiments confirmed the hypothesis on highly correlated sensors and the method’s effectiveness in detecting failures, as demonstrated by 13 False Negatives recorded during rotor compressor speed analysis, highlights the system’s ability to minimize missed detections.

## Figures and Tables

**Table 1:** The training dataset fragment obtained during flight tests for the TV3-117 engine.

Value	1	…	40	…	94	…	158	…	209	…	235	…	256
*n_TC_*	0.685	…	0.971	…	0.902	…	0.903	…	0.911	…	0.740	…	0.823
*n_FT_*	0.531	…	0.986	…	0.745	…	0.752	…	0.761	…	0.454	…	0.505
$T_{G}^{*}$	0.487	…	0.979	…	0.711	…	0.721	…	0.723	…	0.518	…	0.521

**Table 2:** Calculation results for correlation coefficients among sensor sets.

Sensor Sets	Correlation Coefficient Values	Title 3
*n_TC_* and *n_FT_*	0.876	Strong positive correlation
*n_TC_*, *n_FT_* and $T_{G}^{*}$	0.913	The robust positive correlation
*n_TC_*, *n_FT_*, $T_{G}^{*}$, *T_oil_* and *P_oil_*	0.754	Moderate positive correlation
*n_TC_*, *n_FT_*, $T_{G}^{*}$, *T_oil_*, *P_oil_* and *n_rs_*	0.735	Moderate positive correlation

**Table 3:** Testing results for the developed system (see Figure 10) on an AMD Ryzen 5 5600 processor with 3.5 GHz, 6 cores, and 12 threads.

Cores Number	Threads Number	Time, s	Relative Time, s
1	1	363.73	1.000
	2	191.17	1.644
2	3	138.23	2.711
	4	113.94	3.226
3	5	96.18	3.835
	6	85.88	4.108
4	7	80.92	4.218
	8	73.16	4.332
5	9	70.24	4.425
	10	63.11	4.875
6	11	55.63	5.218
	12	50.02	6.000

**Table 4:** The input data description (fragment).

Time	Sensor Parameter					
	*n_TC_*	*n_FT_*	$T_{G}^{*}$	*T_oil_*	*P_oil_*	*n_rs_*
80	0.876	0.765	0.742	1.000	1.000	0.998
90	0.876	0.765	0.738	0.999	1.000	0.998
100	0.885	0.801	0.750	0.999	0.999	0.997
110	0.883	0.803	0.753	1.000	0.999	0.999
120	0.887	0.797	0.744	1.000	1.000	0.999
…	…	…	…	…	…	…
320	0.792	0.541	0.570	0.998	0.999	0.997

**Table 5:** The semi-physical simulation stands the test results.

Telemetry File Name	Telemetry Readings Numbers	Processing Time, s
N17.dat	2560	17.342
N18.dat	3240	17.342

**Table 6:** Comparative analysis results.

Metric	Proposed Approach	Alternative Approach 1	Alternative Approach 2
Accuracy	0.993	0.981	0.982
Precision	0.987	0.980	0.979
Recall	1.0	1.0	1.0
F1-score	0.993	0.988	0.989

**Table 7:** Comparative analysis results (AUC-ROC analysis).

Metric	Proposed Approach	Alternative Approach 1	Alternative Approach 2
True Positives	99	99	98
True Negatives	1	2	3
False Positives	289	288	285
False Negatives	13	15	18
True Positive Rate	0.822	0.819	0.818
False Positive Rate	0.0111	0.0113	0.0112
False Negative Rate	0.0099	0.0101	0.0101
AUC-ROC	0.823	0.818	0.818

**Table 8:** Comparative analysis results.

Metric	Proposed Approach	Alternative Approach 1	Alternative Approach 2
Accuracy (*Acc*)	0.993	0.981	0.982
Training rate (*Res*)	4 min 13 s (253 s)	5 min 43 s (343 s)	4 min 46 s (286 s)
Efficiency	0.00392	0.00288	0.00343

**Table 9:** Comparative analysis results.

Task	Processing Time, s	Required Performance Estimation, GFLOPS	Description
Monitoring the helicopter TE sensor system under interference conditions.	0.0165	4.107	Ensuring stable operation despite external noise.
Restoring regular sensor readings for helicopter TE.	0.0045	1.219	Effective correction of incorrect data
Prediction for a numerical series value.	0.0050	1.674	Enabling accurate forecasting based on historical data.
Total	0.026	7.0	The developed system demonstrates robust performance, ensuring stable operation under interference, the effective correction of erroneous data, and accurate predicting based on historical trends, making it highly reliable for real-time applications.

**Table 10:** The developed neural network system’ proposed further improvements.

Improvement Proposed	Description
Improve the data preprocessing mechanism.	Develop more adaptive discretization and quantization algorithms to better account for input data features such as noise or outliers; Include additional data cleaning methods, such as outlier filtering, using statistical or machine learning methods.
The network architecture optimization is improved.	Investigate the Transformer’s possibility for time series processing, which can outperform LSTM/GRU when working with long sequences due to the attention mechanism; Add additional regularization layers, such as Dropout or Batch Normalization, to improve the model’s resistance to overfitting.
The correlation analysis accuracy is improved.	Implement dynamic attention mechanisms that automatically identify key time points and sensors with maximum correlation; Use advanced methods of correlation analysis, including nonlinear dependencies, such as mutual information.
Expanding network functionality.	Add the ability to predict not only correlations but also other dependencies, such as time lags or trends; Integrate anomaly models based on autoencoders for more accurate fault detection.
Training optimization.	Develop a hybrid optimizer that combines features of Adam and RMSProp to update weights more accurately and speed up convergence; Include mechanisms for early stopping and dynamic changes in the training rate based on validation metrics.
The system’s interpretability is improved.	Develop visualization modules that display not only the correlation results but also the training dynamics, weights, and activation distribution. Implement network decision explanation tools, such as SHAP or LIME, to analyze the individual input contribution.
Modular integration.	Develop the ability to integrate the system with real devices and cloud platforms for real-time data processing. Create an API for easy interaction with external modules, such as control systems or analytical platforms.
Scalability and resilience.	The computing resources used are optimized, for example, by parallelizing calculations on GPUs or TPUs. Add mechanisms to check and recover from failures to improve the system’s reliability.

## Data Availability

The data are contained within the article.
